# Replacing critical point drying with a low-cost chemical drying provides comparable surface image quality of glandular trichomes from leaves of *Millingtonia hortensis* L. f. in scanning electron micrograph

**DOI:** 10.1186/s42649-020-00035-6

**Published:** 2020-07-17

**Authors:** Raktim Bhattacharya, Sulagna Saha, Olga Kostina, Lyudmila Muravnik, Adinpunya Mitra

**Affiliations:** 1 Natural Product Biotechnology Group, Agricultural and Food Engineering Department, Indian Institute of Technology Kharagpur, Kharagpur, 721302 India; 2 Laboratory of Plant Anatomy and Morphology, Komarov Botanical Institute, Russian Academy of Sciences, Professor Popov Street 2, Saint Petersburg, Russia 197376

**Keywords:** Scanning electron microscope, Critical-point drying, Chemical drying, Hexamethyldisilazane, *Millingtonia hortensis*, Glandular trichomes

## Abstract

Sample preparation including dehydration and drying of samples is the most intricate part of scanning electron microscopy. Most current sample preparation protocols use critical-point drying with liquid carbon dioxide. Very few studies have reported samples that were dried using chemical reagents. In this study, we used hexamethyldisilazane, a chemical drying reagent, to prepare plant samples. As glandular trichomes are among the most fragile and sensitive surface structures found on plants, we used *Millingtonia hortensis* leaf samples as our study materials because they contain abundant glandular trichomes. The results obtained using this new method are identical to those produced via critical-point drying.

## Introduction

Scanning electron microscopy (SEM) is widely used to generate detailed images of the surface morphology of plant samples. The steps required to prepare any tissue for SEM include fixation, dehydration, critical-point drying (CPD), mounting, and coating with gold or palladium to improve the electrical conductivity of tissue samples (Bomblies et al. 2008). While hard structures can usually be air-dried prior to coating with metal for SEM, soft plant tissues must be chemically fixed (hardened), carefully dehydrated, and dried. Simple air drying, even of chemically hardened tissues, can cause collapse and shrinkage (Nation 1983). While the fixation procedure is straightforward and does not require expensive equipment, dehydration needs to be carried out carefully to ensure the preservation of cell structure and to avoid tissue shrinkage (Pathan et al. 2010). Dehydration removes water from tissues. In this process, samples to be examined are exposed to increasing concentrations of graded ethanol, resulting in complete removal of water molecules from the samples (Meek 1976).

Drying is the final preparatory stage of SEM sample preparation. Drying completely removes any intermediate solvents or dehydrating agents from tissues (Meek 1976). Standard drying methods used for SEM sample preparation include critical-point drying and air drying. The most commonly used drying method for preparing biological samples is CPD using liquid carbon dioxide (CO_2_). This method removes liquid from tissues while avoiding surface tension effects, where the transition from liquid to gas at the critical point occurs without an interface as the densities of the liquid and gas are equal to this point (Meek 1976). Thus, CPD in general is the method of choice for drying biological specimens, including trichome analysis of plant tissues (Zuzarte et al. 2010; Livingston et al. 2020), despite longer sample preparation times (Shively and Miller 2009). The major disadvantage of CPD is its cost, as a specialized device is needed for liquid CO_2_ under a vacuum, which can be too expensive for small laboratories on an individual scale. Furthermore, CPD is not a glitch-free method; a minor change in the parameters during CPD may lead to a vacuum (Boyde 1980).

In an earlier study, microorganisms were examined as specimens embedded in a matrix rather than using CPD for bacterial samples. This was because the traditional CPD-based techniques used to prepare bacterial samples often form irregular artifacts, which does not occur with air-dried samples (Nierzwicki-Baur 1986). Schols et al. (2004) reported that using CPD (Balzers CPD 030) produced unsatisfactory results for drying pollen grains collected from herbarium samples. In this study, CPD caused the collapse of pollen grains in all the experimental designs with CPD. A decrease in the quality of pollen grains during sample preparation using CPD was also described in an earlier study (Adams and Morton 1972). Further, it was also reported that CPD can cause thermal and pressure stresses in tissues for an extended period and may extract cellular components from the transitional fluid (Gunning and Crang 1984).

An alternative to CPD for drying plant tissue samples is the use of low-cost chemicals such as hexamethyldisilazane (HMDS) and Peldri II (Zimmer and Peldri 1989). Although only scant information is available on chemical drying of plant tissues for SEM, Peldri II treatment in leaves showed complete removal of epicuticular wax, while CPD and HMDS retained the surface microstructure (Bray et al. 1993; Chissoe et al. 1994; Pathan et al. 2010). Using HMDS to dry biological samples is not new, particularly with animal tissues. Soft tissues being prepared for SEM were dehydrated through a graded ethanol series, immersed in HMDS, and air-dried without critical-point drying (Nation 1983). The reduced surface tension of HMDS strengthened the samples during drying and possibly reduced fracturing of collapsing animal tissues (Nation 1983). Several studies were conducted with HMDS as a chemical drying reagent, but all used animal and human tissues (Braet et al. 1997; Shively and Miller 2009). Information on HMDS as a chemical agent for drying plant tissues after fixation for SEM analysis is scant (Bray et al. 1993). These reports did not recommend HMDS as a chemical drying agent to study delicate surface structures via SEM (Bray et al. 1993). Chissoe et al. (1994) for the first time promoted HMDS as a drying reagent to overcome problems associated with drying pollen grains via CPD for SEM analysis. In a subsequent study, HMDS was also used to prepare microbial samples from anaerobic biofilms for SEM analysis. A comparison of CPD and HMDS-dried samples demonstrated that HMDS did not disrupt cell structures of microorganisms as evidenced with CPD (Araujo et al. 2003). Apart from chemical drying, some researchers use air-drying, which often produces tissue distortions (Zimmer and Peldri 1989). In plants, glandular trichomes are considered among the most delicate surface structures (Muravnik et al. 2016). This study reports our findings on SEM analysis of leaf glandular trichomes from *Millingtonia hortensis*, a Bignoniaceae tree species prevalent throughout southeast Asia, to reassess the CPD, HMDS, and air-drying methods.

## Materials and methods

Small leaves of *M. hortensis* were used as our study material. They were collected fresh from greenhouse-grown plants at Komarov Botanical Institute in St. Petersburg, Russia. In India, leaf samples were collected fresh from field-grown plants at the Indian Institute of Technology Kharagpur. During the sample collection, the leaves’ developmental stages were assessed and were same in both cases.

After collection, the samples were immediately immersed in a fixative solution containing 2.5% (v/v) glutaraldehyde and 4% (v/v) paraformaldehyde in 0.1 M of phosphate buffer and placed under a vacuum until all the samples sank to the bottom of the vials. After the first round of fixation, half of the samples were post-fixed in 2% (v/v) osmium tetroxide (OsO_4_) (Sigma Aldrich) in 0.1 M of phosphate buffer at 4 °C overnight. The other half of the samples were dehydrated in 30%, 50%, 70%, 80%, and 90% ethanol (for 10 min each) and two times in 95% ethanol (20 min each) in succession at room temperature. The samples were then maintained in a mixture of 95% ethanol and isoamyl acetate (1:1) for 10 min and in pure isoamyl acetate for 15 min. After removing isoamyl acetate, the samples were placed on a sample holder for critical-point drying in a Hitachi HCP-2 critical-point dryer (Hitachi, Japan) according to the method described by Muravnik et al. (2016). The samples that were maintained overnight in OsO_4_ for secondary fixation were treated in the same way (as previously described) on the next day. Both types of samples (treated and untreated with OsO_4_) were sputter-coated with a thin layer of gold and viewed under a JEOL JSM-6390 (JEOL, Japan) scanning electron microscope at an accelerating voltage of 7 kV in the laboratory at Komarov Botanical Institute. Digital images were produced using the microscope’s control program (Muravnik et al. 2016).

For chemical drying, the same protocol was followed until the isoamyl acetate stage and then the samples (treated and untreated with OsO_4_) were maintained separately in HMDS for 5 min at room temperature. The samples were then dried in a desiccator for 30 min and sputter-coated with gold. The samples were viewed under a ZEISS EVO 60 (Carl ZEISS SMT, Germany) scanning electron microscope at an accelerating voltage of 20 kV in the laboratory at the Central Research Facility, Indian Institute of Technology Kharagpur. The microscope’s control program was used to generate digital images.

For air drying, the samples were fixed and then subjected to ethanol dehydration as previously described. After the final round of dehydration with 95% ethanol, the samples were maintained open overnight to ensure the complete evaporation of the ethanol and proper air drying. The air-dried samples were viewed under a ZEISS EVO 60 (Carl Zeiss SMT, Germany) scanning electron microscope at an accelerating voltage of 20 kV after sputter-coating with gold in the laboratory at the Central Research Facility, Indian Institute of Technology Kharagpur. The microscope’s control program was used to generate digital images.

## Results and discussion

**Fig. 1:**
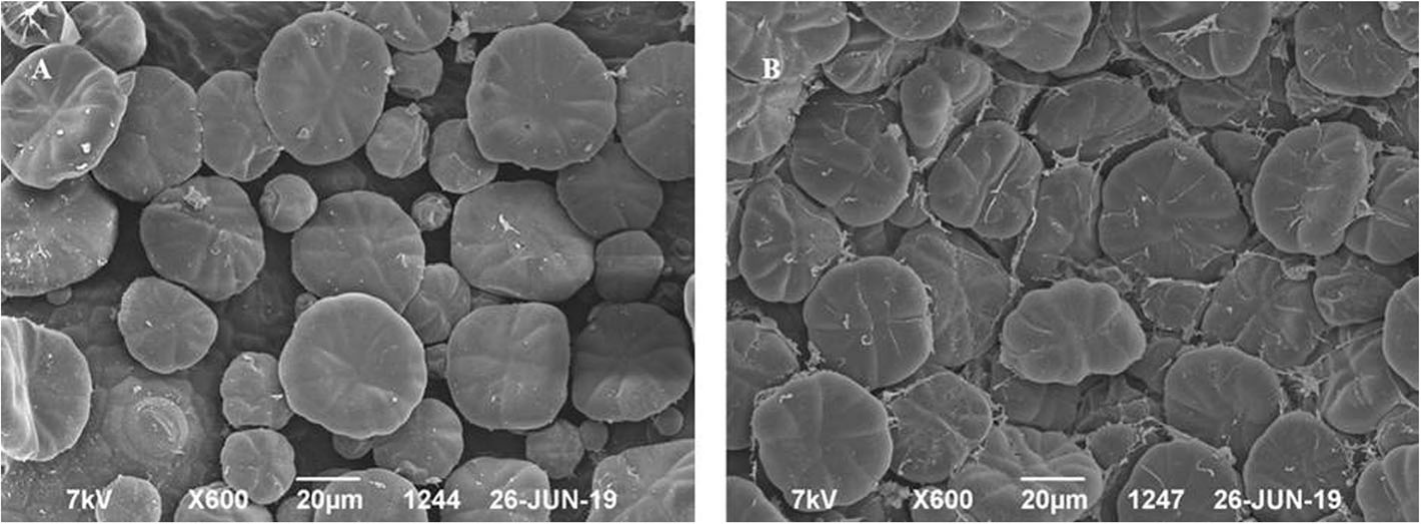
Scanning electron micrograph of the *M. hortensis* leaf surface after critical-point drying (**a**) without OsO_4_ and (**b**) with OsO_4_. Imaged at 7.00 kV with a magnification of X600. Scale bar represents 20 μm

**Fig. 2:**
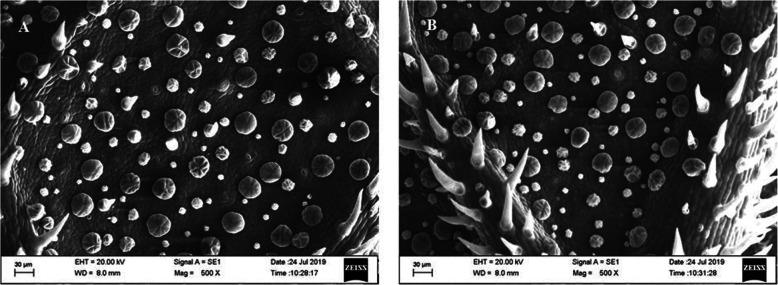
Scanning electron micrograph of the *M. hortensis* leaf surface after HMDS-based drying (**a**) without OsO_4_ and (**b**) with OsO_4_. Imaged at 20.00 kV with a magnification of X500. Scale bar represents 30 μm

**Fig. 3:**
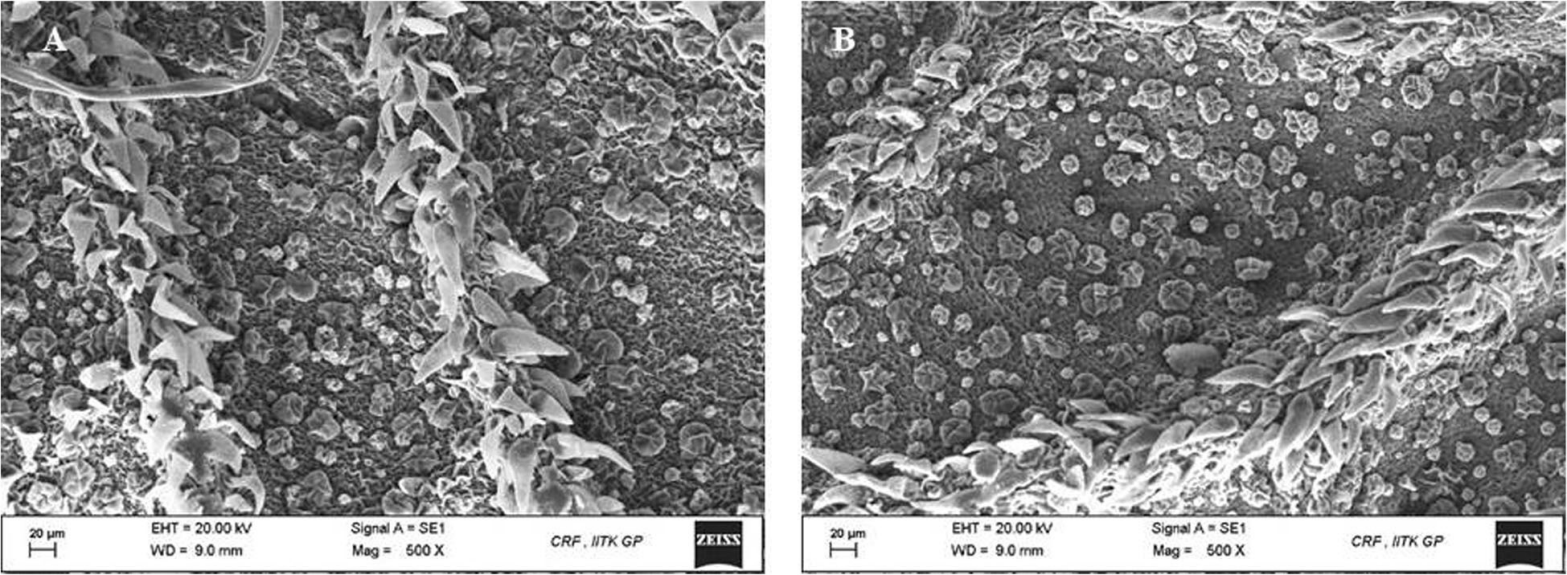
Scanning electron micrograph of the *M. hortensis* leaf surface after air drying (**a**) without OsO_4_, and (**b**) with OsO_4_. Imaged at 20.00 kV with a magnification of X500. Scale bar represents 20 μm

Both the CPD- (Fig. 1) and HMDS-treated (Fig. 2) samples showed an equal range of preservation in trichome architecture and morphology. The mechanism of action of HDMS on biological tissues is unclear. This reagent is often used in gas chromatography to produce silylation of nonvolatile compounds such as sugars, amino acids, and alcohols (Nation 1983). The combined properties of low surface tension and cross-linking potential are likely the reasons for its suitability as chemical drying agent for biological tissues. However, in the air-dried samples, artifacts occurred due to tissue shrinkage. Both the OsO_4_-treated and OsO_4_-untreated samples showed almost identical tissue fixation. But the samples treated with OsO_4_ as the post-fixative agent demonstrated slightly better tissue preservation and image contrast than those that were not post-fixed with OsO_4_. As shown in Fig. 3, the air-dried samples were distorted. Drying-induced artifacts were also evident in the images, rendering the effects of OsO_4_ negligible. The images of the air-dried samples were not comparable with the CPD- and HMDS-based methods as the samples underwent greater structural distortion than the other two methods. As previously mentioned, glandular trichomes are very difficult to image due to their extremely delicate structures. Earlier chemical drying attempts also failed to preserve the trichome structure (Zimmer and Peldri 1989). However, in our method, when using HMDS, fine structures of glandular trichomes in the young leaves were successfully preserved and no marked differences could be distinguished between the images obtained using CPD (Fig. 1) and HMDS (Fig. 2).

Avoiding CPD means no need to invest in costly instruments by individual laboratories working on a small scale. HMDS enables small laboratories to prepare samples in their labs and ensure a single trip to the SEM facility. However, the chemical properties of HMDS have safety concerns. As it is a corrosive substance, gloves and masks are recommended when handling HMDS inside fume hoods. HMDS is not the only reagent used in SEM sample preparation; many other chemicals used for this purpose are corrosive, such as isoamyl acetate, glutaraldehyde, and OsO_4_. It can be maintained normally at room temperature in an amber bottle, and because the boiling point is quite high (125 °C), HMDS can withstand significant temperature variations. However, although air drying is a low-cost method, it is unable to preserve the natural structure of trichomes. Therefore, HMDS should be considered a substitute for air drying and CPD when preparing plant samples for SEM analysis.

**Fig. 4:**
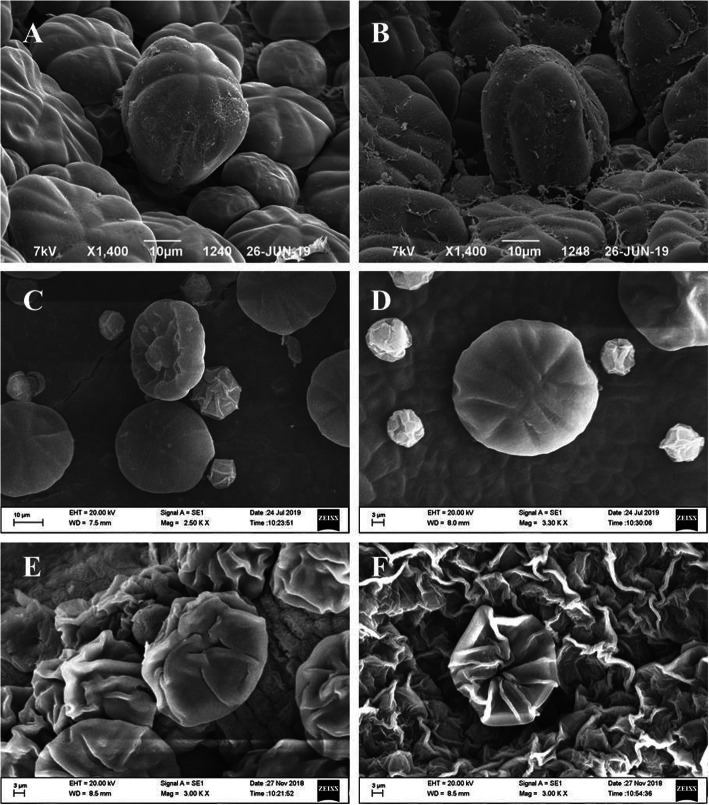
Scanning electron micrograph of the *M. hortensis* leaf surface after CPD (**a** and **b**), HMDS (**c** and **d**), and air drying (**e** and **f**). All the samples were treated with OsO_4_ as a secondary fixative while maintaining all of the sample preparation parameters, except for the final drying process. Scale bars represent 10 μm in **a**, **b**, and **c** and 3 μm in **d**, **e**, and **f**

As shown in the highly magnified image (Fig. 4), it is evident that the HMDS-based drying produced results comparable (Fig. 4c and d) to CPD-based drying (Fig. 4a and b). However, both the CPD and HMDS-based drying methods produced SEM images with better clarity than air drying (Fig. 4e and f).

Some amount of charging was observed in the HMDS-treated samples (Fig. 4c and d) and air-dried samples (Fig. 4e and f). The air-dried samples showed complete destruction of their natural structures that may have caused uneven gold coating, leading to charging of the samples. However, we are unable to provide any conclusive remarks about the charging of the HMDS-treated samples. This is because the HMDS-treated samples demonstrated a minor amount of charging, and unlike air drying, there was no major structural distortion of the samples. As charging can be due to many reasons, including the gold-coating time, the coating’s thickness, sample dehydration, and the electron beam energy, among other factors, it is difficult to ascertain the cause of charging. In the literature survey, the authors found that samples prepared using CPD also demonstrated a considerable amount of charging (Zuzarte et al. 2010). Thus, in our opinion, the minor charging in the HMDS-treated samples may not have been due to the drying method used.

## Conclusion

This study demonstrated that the quality of preservation using CPD and HMDS was identical. Further, preservation of delicate structures such as glandular trichome indicates that HMDS can be widely used in different types of plant materials. HMDS-mediated drying should be of interest to plant biologists, as using HMDS instead of CPD saves considerable sample preparation time.

## Data Availability

Please contact the corresponding author for data availability.

## Abbreviations

SEM: Scanning electron microscopy
CPD: Critical-point drying
CO_2_: Carbon dioxide
HMDS: Hexamethyldisilazane
OsO_4_: Osmium tetroxide
